# Key gene network related to primary ciliary dyskinesia in hippocampus of patients with Alzheimer’s disease revealed by weighted gene co-expression network analysis

**DOI:** 10.1186/s12883-022-02724-z

**Published:** 2022-05-30

**Authors:** Pengcheng Xia, Jing Chen, Xiaohui Bai, Ming Li, Le Wang, Zhiming Lu

**Affiliations:** 1 Department of Clinical Laboratory Medicine, Shandong Provincial Hospital Affiliated to Shandong First Medical University, Jinan, Shandong China; 2 Discipline of Anatomy and Pathology, Shandong First Medical University, Jinan, Shandong China; 3 School of Medicine, Shandong University, Jinan, Shandong China

**Keywords:** Alzheimer’s disease, Primary ciliary dyskinesia, WGCNA, Functional enrichment analysis, Protein-protein interaction network, hub gene

## Abstract

**Background:**

Alzheimer’s disease (AD) is closely related to aging, showing an increasing incidence rate for years. As one of the main brain regions involved in AD, hippocampus has been extensively studied due to its association with many human diseases. However, little is known about its association with primary ciliary dyskinesia (PCD).

**Material and Methods:**

The microarray data of hippocampus on AD were retrieved from the Gene Expression Omnibus (GEO) database to construct the co-expression network by weighted gene co-expression network analysis (WGCNA). The gene network modules associated with AD screened with the common genes were further annotated based on Gene Ontology (GO) database and enriched based on the Kyoto Encyclopedia of Genes and Genomes (KEGG) database. The protein-protein interaction (PPI) network was constructed based on STRING database to identify the hub genes in the network.

**Results:**

Genes involved in PCD were identified in the hippocampus of AD patients. Functional analysis revealed that these genes were mainly enriched in ciliary tissue, ciliary assembly, axoneme assembly, ciliary movement, microtubule based process, microtubule based movement, organelle assembly, axoneme dynamin complex, cell projection tissue, and microtubule cytoskeleton tissue. A total of 20 central genes, e.g., *DYNLRB2, ZMYND10, DRC1, DNAH5, WDR16, TTC25,* and *ARMC4* were identified as hub genes related to PCD in hippocampus of AD patients.

**Conclusion:**

Our study demonstrated that AD and PCD have common metabolic pathways. These common pathways provide novel evidence for further investigation of the pathophysiological mechanism and the hub genes suggest new therapeutic targets for the diagnosis and treatment of AD and PCD.

**Subjects:**

Bioinformatics, Cell Biology, Molecular Biology, Neurology.

**Supplementary Information:**

The online version contains supplementary material available at 10.1186/s12883-022-02724-z.

## Introduction

As the most common progressive neurodegenerative disorder associated with aging [1], Alzheimer’s disease (AD) is the main cause of dementia in the elderly people [2]. Studies have found that AD is closely related to a variety of factors, such as oxidative stress [3], autophagy dysfunction [4], and diabetes [5]. Although the studies on AD have advanced greatly, the number of AD patients worldwide is still increasing dramatically. It is predicted that by 2050, there may be four times more patients with AD [6], causing significant inconvenience to patients and financial burden to the society [7]. Although some drugs have been developed to target intermediate products, e.g., aggregated Amyloid β-protein (A-β), involved in the known developmental mechanisms of AD, almost all drugs fail to significantly improve patients’ symptoms [8]. Hippocampus is among the most important brain structures involved in memory, and is a critical site of pathogenesis in dementing illnesses such as AD [9]. The hippocampal formation was essential for the proper functioning of spatial and episodic memory in the early stages of AD [10]. There are also studies on the correlation between hippocampal volume and AD, however, no explicit relationship was revealed [11]. In animal experiments, shRNA silenced Egr-1 in the hippocampus and improved the cognition of 3xTG AD mouse model [12]. Adult hippocampal neurogenesis (AHN) impairment contributes significantly to the cognitive decline in patients of AD [13], More importantly, hippocampus is also the main location for A-β deposition [14]. Therefore, it is crucial to understand the pathological status of hippocampus in AD patients in order to study the mechanisms of AD-related memory impairments [15].

Cilia are dynamic microtubule-based organelles present on the surface of many types of eukaryotic cells. Cilia related defects underlie a growing list of human disorders, collectively called ciliopathies, with overlapping phenotypes such as developmental delays and cognitive and memory deficits [16]. It has been reported that Serotonin 5-HT6 receptors affect cognition in a mouse model of AD by regulating cilia function [17]. Microtubules are an important part of cilia, playing an important role in cell division (i.e., beating of cilia and flagella) and intracellular transport [18].

Furthermore, aberrant interaction between the microtubule-associated protein Tau and the filamentous actin is connected to synaptic impairment in AD [19]. PCD is the most representative disease of ciliary dysfunction. It is a rare hereditary disease characterized by abnormal movement of cilia in human body [20]. When the cilia function normally, they beat together, helping to push the mucus through the respiratory system to the throat area, ultimately expelled by coughing. This process is very important for the human body to resist infection [21]. Because cilia play important roles in both AD and PCD, we hypothesized that AD and PCD share similar pathogenesis. It has been reported that there are more than 20 kinds of ultrastructural abnormalities in cilia, most of which are dynein arm defects and microtubule defects. Next generation sequencing has enhanced the gene identification, and mutations in more than 40 genes have been reported to cause PCD, with many other genes likely to be discovered [22]. Although studies have demonstrated that genes related to PCD such as dynein axonemal intermediate chain 1 (*DNAI1*) play an important role in AD [23], no studies in-depth have been conducted. To date, studies on the association between AD and PCD are sparse. Therefore, it is necessary to study the relationship between AD and PCD, especially at the molecular and genetic levels.

To date, the analysis of weighted gene co-expression network (WGCNA) is a well-established advanced method applied to investigate the molecular mechanism of genes and reconstruct gene co-expression network through transforming the adjacency matrix into a topological overlap matrix [24]. In this study, we have identified the gene set related to PCD in the hippocampus of AD patients based on WGCNA analysis and further investigated the association between PCD and AD.

## Materials & Methods

### Data preparation

Gene expression profiles of AD were downloaded from Gene Expression Omnibus (GEO) database (https://www.ncbi.nlm.nih.gov/geo/) [25]. The dataset of GSE48350 [26] based on GPL570 [HG-U133_Plus_2] Affymetrix Human Genome U133 Plus 2.0 Array contained microarray data from normal controls (aged 20–99 years) and AD cases from 4 brain regions, including hippocampus, entorhinal cortex, superior frontal cortex, and post-central gyrus. Expression levels of synaptic and immune related genes were assessed to investigate the age-related changes, AD-related changes, and region-specific patterns of changes. A total of 19 samples of AD and 43 samples of normal controls of hippocampus tissues were included in this study. To validate the results of our analysis, the transcriptomic profiles of the human AD hippocampus GSE36980, GSE1297, GSE28146, GSE29378 datasets were used.

### Differential gene analysis

All differential gene analyses were performed by R foundation for statistical computing (2020) version 4.0.3. The dataset used for differential gene analysis was retrieved from GEO database with the format of MINIML. Limma package (version: 3.40.2) of R software was used to investigate the differential expression of mRNAs. The effect of remove the batch was performed by using the ‘remove Batch Effect’ command in Limma package. The adjusted *P*-value was analyzed to correct the false positive results in GEO datasets. The parameters “Adjusted *P* < 0.05 and Log (Fold Change) >1 or Log (Fold Change) < −1” were defined as the thresholds for the screening of differential expression of mRNAs. The box plot was generated by the R software package ggplot2. The heatmap was displayed by the R software package pheatmap.

### GO annotation and KEGG pathway enrichment analysis

To further confirm the underlying functions of potential target genes identified by differential gene analysis, these genes were analyzed by functional enrichment based on Gene Ontology (GO) and Kyoto Encyclopedia of Genes and Genomes (KEGG) databases [27]. GO is a widely used tool for annotating genes with functions in three categories, including molecular function (MF), biological pathways (BP), and cellular components (CC). KEGG is a practical resource for analytical study of gene functions and associated high-level genome functional information. To better understand the function of mRNAs, Cluster Profiler package (version: 3.18.0) in R was employed to further analyze the GO functions of potential target genes and the enrichment of the KEGG pathway. FDR is a common term in statistics. It is translated as false discovery rate. The expected value of the ratio. In the enrichment results, *P* < 0.05 or false discovery rate (FDR) < 0.05 was considered to be enriched to a meaningful pathway.

### Gene set enrichment analysis

Gene Set Enrichment Analysis (GSEA, https://www.gseamsigdb.org/gsea/index.jsp) was applied to identify the significant pathways in dataset GSE48350. This method determines whether a priori defined set of genes show statistically significant differences or not between two biological states (e.g. phenotypes) [28]. The coefficients of Spearman correlation between genes and sample labels were defined as the weight of genes [29]. Statistical significance was assessed by comparing the enrichment score with the enrichment results generated from 1000 random permutations of the gene sets to obtain nominal *P* values. The significant level of pathways was determined by the levels of normalized enrichment score (NES) ≥ 1.0, FDR ≤ 0.25, and *P* ≤ 0.05.

### Weighted gene co-expression network analysis

Weighted Gene Co-expression Network and co-expression modules were constructed by Weighted Gene Co-expression Network Analysis (WGCNA), which was performed using the WGCNA package in R [30]. The hippocampus regions of the microarray data of GSE48350 were applied as a primary source of data for the WGCNA analysis. The network construction started by calculating robust correlations between all genes across in all relevant samples. The correlation adjacency matrix was increased to the power β = 18 based on scale-free topology criterion. The power parameter was selected to amplify the strong connections between genes and to penalize the weak connections. The first principal component was considered as the module eigen gene (ME), representing the highest percent of variance for all the genes in a module. Module membership (kME) measured the correlations between each gene and each ME. The within-module connectivity (kin) for each gene was determined by summing the connectivity of that gene with each of the other gene set in the same module [31, 32], showing significant correlations with MEs and high within-module connectivity, and were considered as hub genes of the modules. The hub genes were verified by using Cytoscape’s cytoHubba plugin [33]. In order to analyze the correlation between module and phenotype, we transformed the classified variables into numerical variables. Specifically, female was defined as 0 and male as 1; non-AD patients under 80 years old were defined as 0 and non-AD patients above 80 years old as 0.5; and AD positive as 1. In this study, a total of 3 phenotypes were analyzed using Spearman method to calculate the correlation.

### Protein-Protein Interaction (PPI) analysis

All common genes from the selected modules were further analyzed by the online Search Tool for the Retrieval of Interacting Genes (STRING) database (Version 11.0; http://string-db.org/) to establish the network through protein-protein interaction (PPI) analysis [34]. A combined score of more than 0.4 was applied to build the PPI network, which was visualized by the Cytoscape software (version 3.8.2, http://cytoscape.org/) [35]. The common genes in networks were screened by the degree of the gene nodes. The genes with the most interactions were considered as hub genes, which may play important roles in the disease’s pathogenesis.

### Gene analysis by Venn diagram

Venn diagrams are used to show logical connections between different groups of things (sets). In addition to Hub genes, the other two sites involved are Genecards database (https://www.genecards.org) and Disgenet database (https://www.disgenet.org/).

### Gene interaction heatmap

Gene correlation can be used to observe the relationship between different genes and to study the relationship between an unknown gene and a known gene in order to predict the function of the unknown gene. The negative and the positive correlations between two genes suggest that these genes may cooperate or antagonize with each other, respectively.

### Prediction of miRNAs of hub genes

miRecords, which is an integrated resource of 11 established miRNA target prediction programs [36], was used to identify the stem loop miRNAs of hub genes. The miRNAs predicted by at least four programs were regarded as the stem loop miRNAs of hub genes. The predicted results are imported into Cytoscape software for a more intuitive display.

### Statistical analysis

GraphPad Prism (version 8.0.0) was utilized to perform the statistical analysis. The normality test and homogeneity of variance test were performed on data extracted from GEO datasets. Data that passed these two tests underwent t-testing for comparisons between two groups. Spearman test was used to investigate the correlation between module and phenotype in WGCNA analysis. The Gene Interaction Heatmap was used to assess the correlation of gene set between AD and PCD. Venn diagram was applied to find the similarity between data sets. *P* values less than 0.05 were considered statistically significant.

## Results

### Screening of differentially expressed genes (DEGs)

**Fig. 1:**
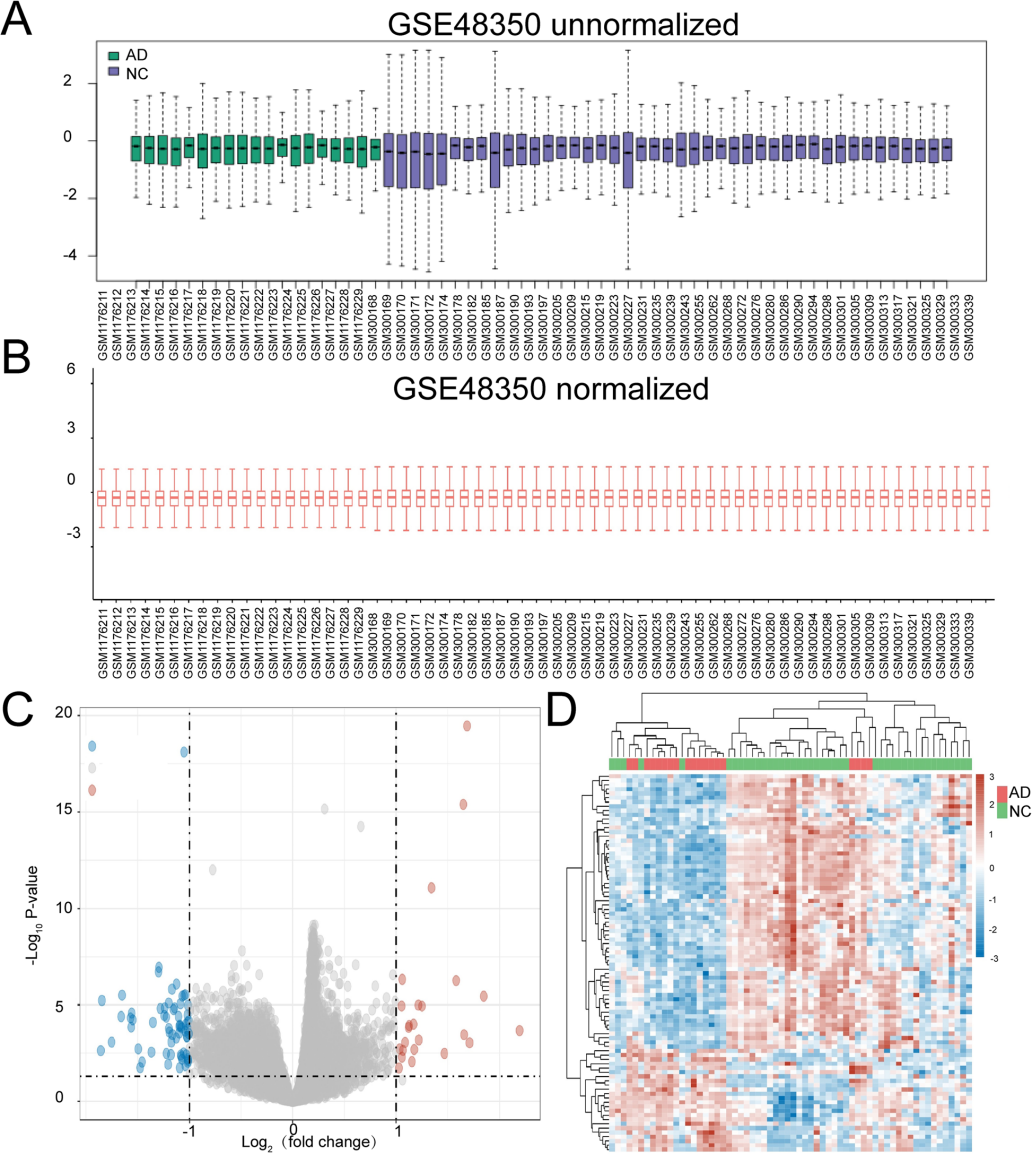
Differential gene analysis of GSE48350. **A** Box plots based on unnormalized data. **B** Box plots based on normalized data. **C** Volcano plots constructed using fold-change values and adjusted *P* values. The red dots represent the over-expressed mRNAs and the blue dots indicate the down-expressed mRNAs with statistical significance. **D** Hierarchical clustering analysis of differentially expressed mRNAs between AD and normal tissues

The gene expression data (GSE48350) on 19 patients with AD were compared with those of 43 control samples (CTs) from GEO database (Tables S1). All samples were hippocampus tissues. After the run of ‘remove Batch Effect’ command in Limma package, the expression level of genes was basically at the same level, which was selected for downstream difference analysis (Fig. 1A, B; Table S2). Based on the cutoff criteria, a total of 88 DEGs (24 up-regulated and 64 down-regulated) were identified in AD samples (Fig. 1B). Heatmap was used to show gene expression levels (Fig. 1C). According to the absolute value of Log, top 10 genes included *LTF, SLC17A6, CFAP126, TMEM155, CALB1, SLC47A2, ANKIB1, NWD2, CP,* and *MRAP2.*

### GO Annotation and KEGG pathway enrichment analysis

**Fig. 2:**
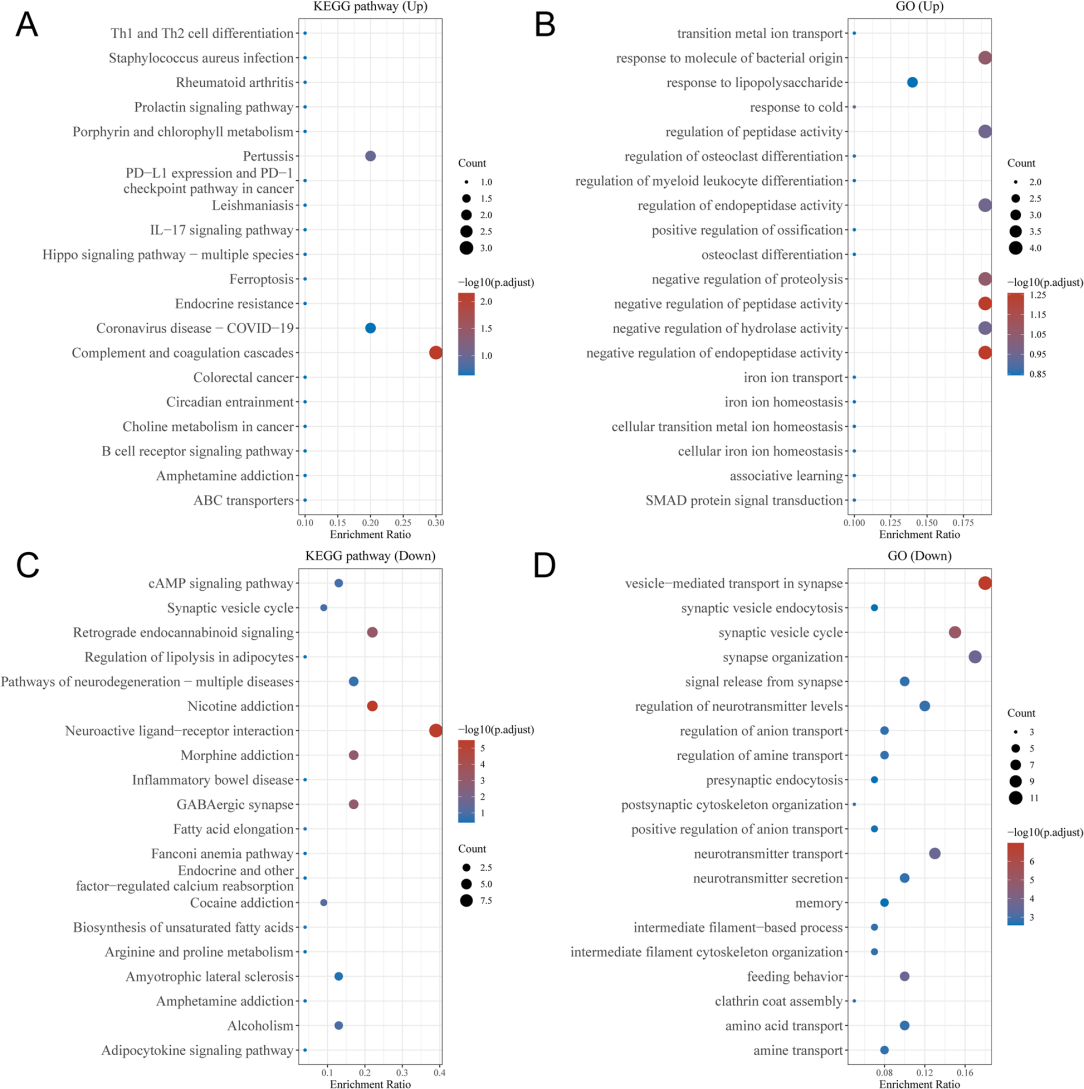
The enriched KEGG signaling pathways and GO analysis demonstrating the primary biological functions of major potential mRNAs. **A** The KEGG pathway of up-regulated genes. **B** The GO function of up-regulated genes. **C** The KEGG pathway of down-regulated genes. **D** The GO function of down-regulated genes

To further confirm the underlying functions of potential target genes, DEGs were analyzed by functional enrichment (Fig. 2, Table S3). The up-regulated genes were significantly enriched in KEGG pathways of complement and coagulation cascades and pertussis, and in the GO functions of negative regulation of endopeptidase activity, negative regulation of peptidase activity, response to molecule of bacterial origin, and negative regulation of proteolysis. The down-regulated genes were significantly enriched in the KEGG pathways of nicotine addiction and neuroactive ligand-receptor interaction, and in the GO functions of vesicle-mediated transport in synapse, synaptic vesicle cycle, neurotransmitter transport, and synapse organization.

### Gene Set Enrichment (GSEA) Analysis

**Fig. 3:**
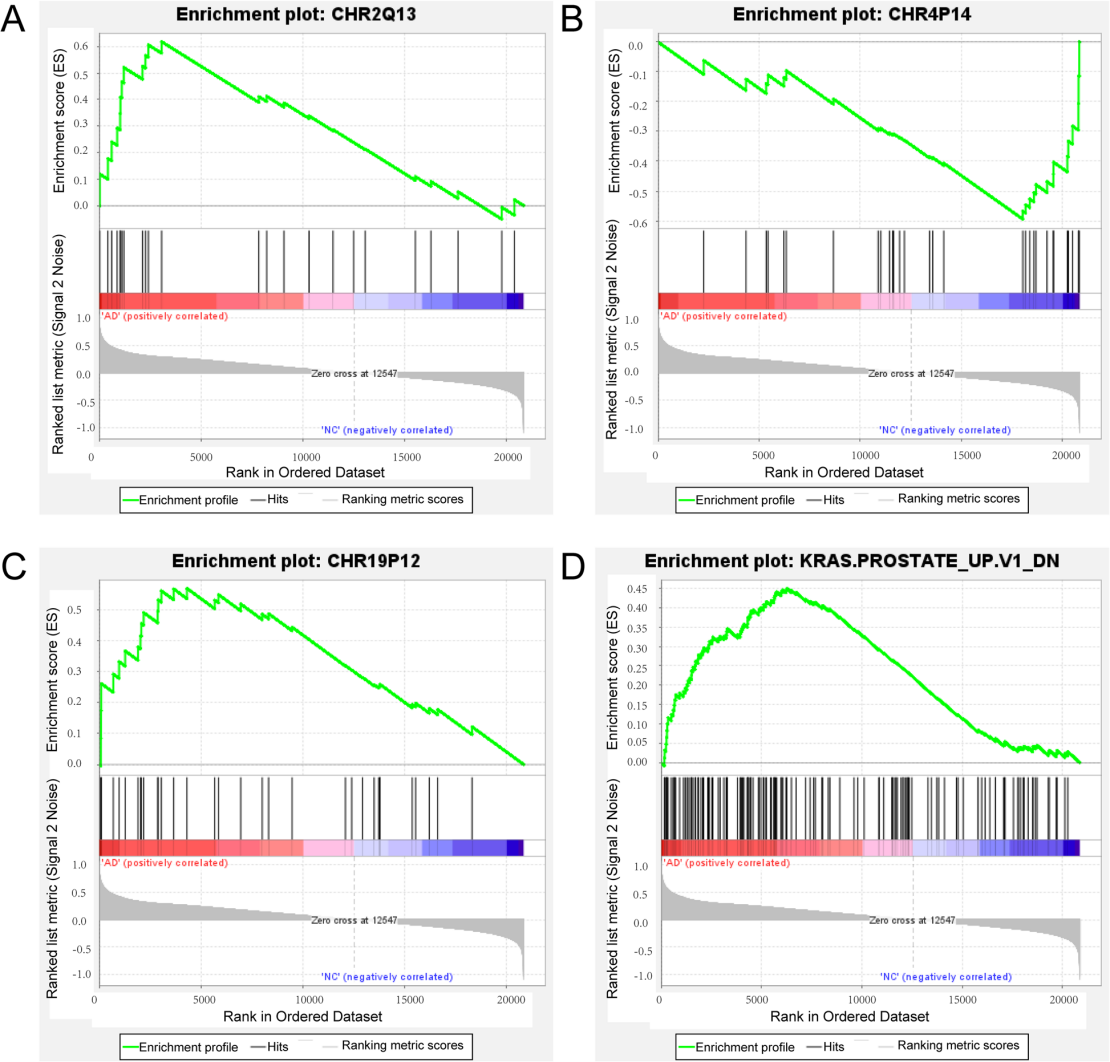
Gene Set Enrichment Analysis (GSEA) of all genes in the array showing the results of the top four GSEA terms based on normalized enrichment scores in the AD. **A** Enrichment plot of *CHR2Q13* (NES = 1.9267, nominal *p*-value = 0.0, FDR q-value = 0.0867). **B** Enrichment plot of *CHR4P14* (NES = 1.9218, nominal p-value = 0.0036, FDR q-value = 0.0121). **C** Enrichment plot of *CHR19P12* (NES = 1.7553, nominal p-value = 0.0154, FDR q-value = 0.2317). **D** Enrichment plot of *KRAS.PROSTATE_UP.V1_DN* (NES = 1.8564, nominal p-value = 0.0, FDR q-value = 0.0482).NES, Normalized Enrichment Score; AD, Alzheimer’s disease; CN, cognitively normal

GSEA is a computational method that determines whether an a priori defined set of genes shows statistically significant, concordant differences between two biological states. We further analyzed the microarray data with the software ‘Gene Set Enrichment Analysis’ [28, 37]. Three up-regulated gene sets in AD including *CHR2Q13*, *CHR19P12*, and *KRAS.PROSTATE_UP. V1_DN* and one down-regulated gene set *CHR4P14* were justified. Three positional gene sets (i.e., *CHR2Q13*, *CHR19P12*, and *CHR4P14*) were enriched in different locations of different chromosomes. *CHR2Q13* is a gene set containing Ensembl 103 genes in cytogenetic band *CHR2Q13*, *CHR19P12* and *CHR4P14* are identical to it. As the oncogenic signature gene set, the *KRAS.PROSTATE_UP. V1_DN* could be down-regulated in epithelial prostate cancer cell lines over-expressing an oncogenic form of KRAS gene. No pathway associated with PCD was identified (Fig. 3, Figs. S1, S2, S3 and S4).

### WGCNA and key module identification

**Fig. 4:**
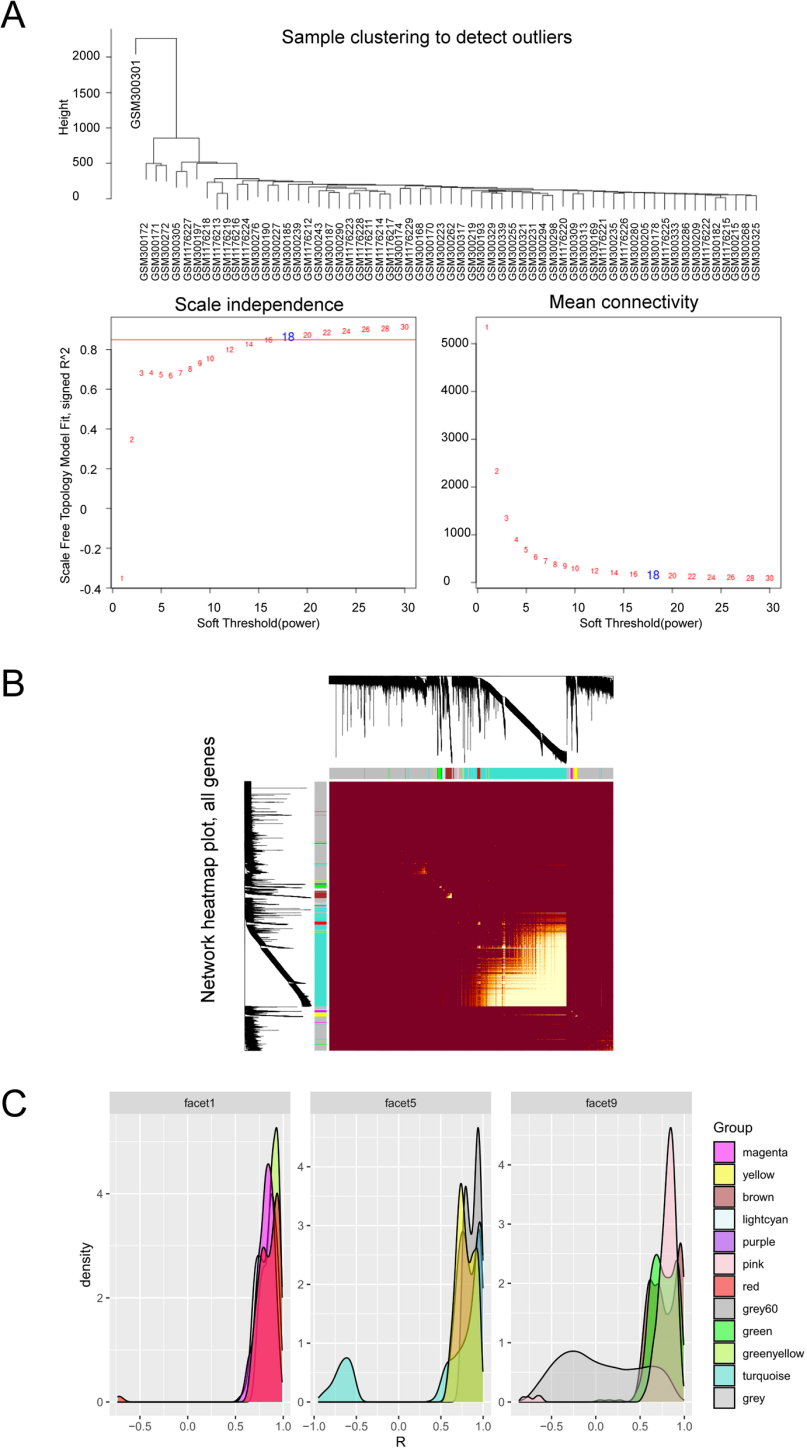
Cluster dendrogram of genes identified by WGCNA. **A** Clustering of samples to detect outliers. Scale-free topology model (left) and mean connectivity (right) were applied to identify the soft-thresholding power. The power selected is 18. **B** Tom diagram of module relationship. **C** The relationship between modules and genes in the modules, the horizontal axis represented the correlation coefficient of gene and module, which was mainly used to observe the distribution of correlation coefficient between gene expression in each module and within the module

**Fig. 5:**
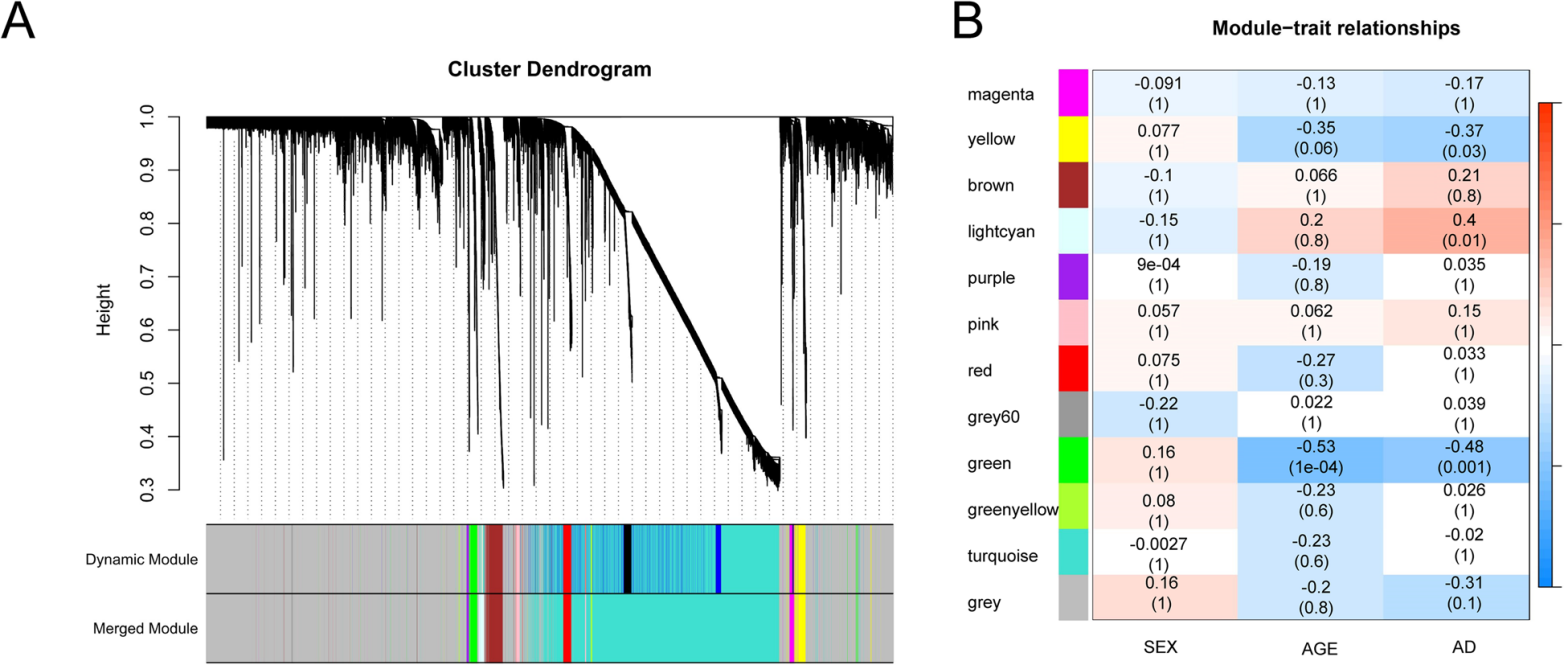
Key modules correlated with AD identified by WGCNA. **A** Clustering of all modules. The red line indicates the height cutoff (0.25). **B** Heatmap showing the relationships between different modules and clinical traits. Non-clustering DEGs in the grey module

All genes in the array were used to conduct WGCNA (Figs. 4 and 5). By setting the soft-threshold power as 18 (scalefree R^2^ = 0.9, blockSize = 7000, minModuleSize = 20, deepSplit = 2, mergeCutHeight = 0.25, hub_cut = 0.9, net_threshold = 0, slope = − 0.96; Fig. 4A), we acquired a total of 12 modules (Fig. 5A; Table S4). The Tom diagram of the relationship between gene clustering and modules in each module of WGCNA was shown in Fig. 4B. The relationship between modules and genes in the modules was shown in Fig. 4C. The correlation coefficient between grey module and gene expression in the module was the lowest. The number of genes in each module was provided in Table 1. Based on the heatmap of module-trait correlations, the key module containing a total of 160 genes was identified as the most positively correlated with AD (correlation coefficient = 0.40, *P* = 0.01; Fig. 5B).

**Table 1:** Module and the number of genes in each module

The color of module	Number of genes in the module
Grey60	27
Greenyellow	71
Purple	74
Magenta	154
Lightcyan	160
Pink	174
Red	266
Green	302
Yellow	343
Brown	551
Turquoise	7090
Grey	11,337

### Selection of hub genes

**Fig. 6:**
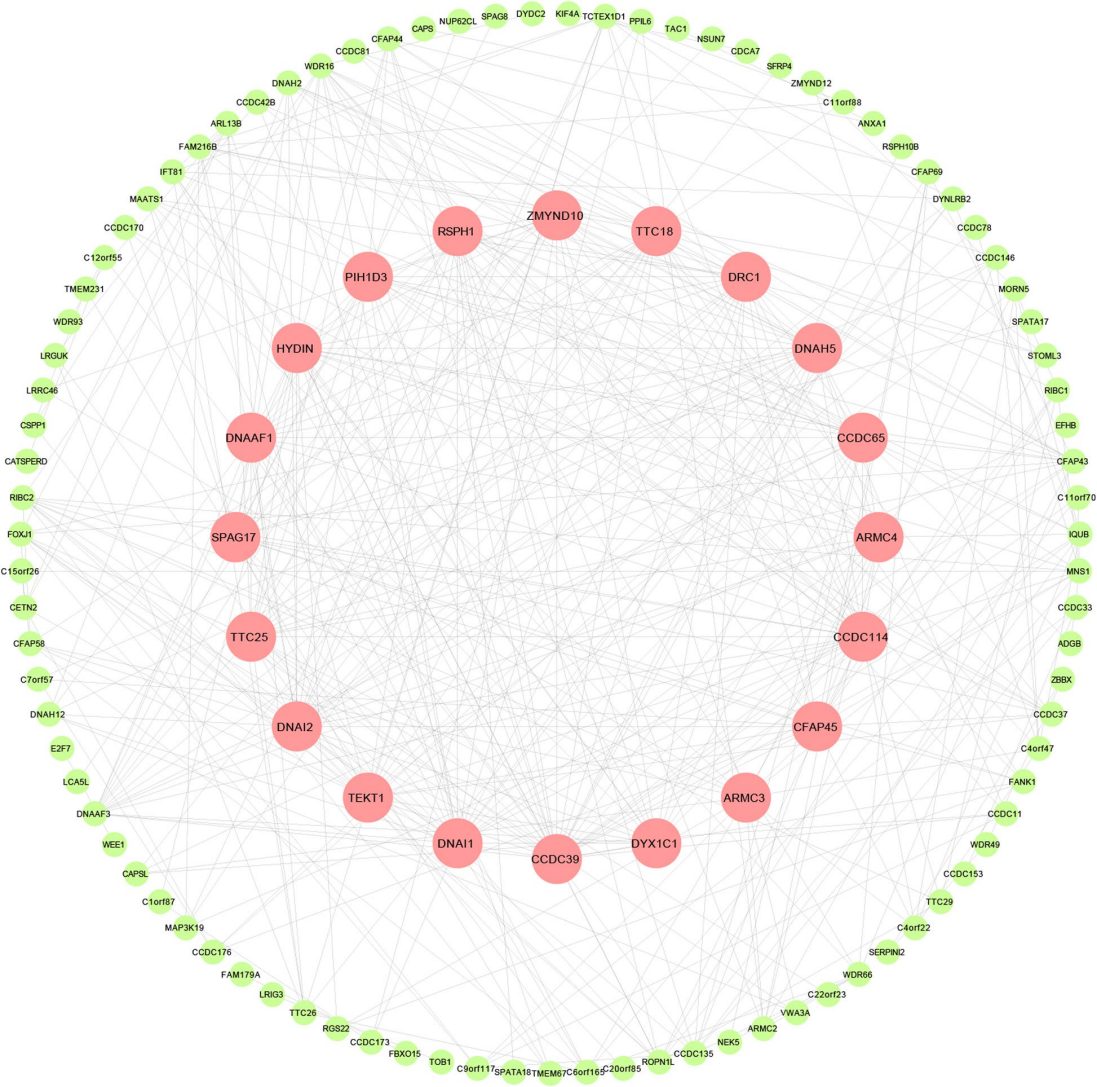
Hub gene set of the key module and interaction network of genes in the key module. Green circles represent genes in the lightcyan module. Red circles represent hub genes in the module

All the common genes from the selected modules were further analyzed by the online STRING database to construct the network. After the PPI analysis, cytoscape was used for visualization and the plug-in cytohubba was used to screen hub genes. The top 20 genes identified were defined as hub genes (Fig. 6; Table 2).

**Table 2:** The Hub genes related to PCD

Gene	Full Name	DPI	Score
*DNAI1*	dynein axonemal intermediate chain 1	0.692	0.6
*DNAAF3*	dynein axonemal assembly factor 3	0.731	0.59
*CCDC114*	coiled-coil domain containing 114	0.5	0.53
*CCDC65*	coiled-coil domain containing 65	0.5	0.52
*DNAH5*	dynein axonemal heavy chain 5	0.692	0.4
*RSPH1*	radial spoke head component 1	0.5	0.36
*CCDC39*	coiled-coil domain containing 39	0.615	0.35
*ZMYND10*	zinc finger MYND-type containing 10	0.731	0.35
*DNAI2*	dynein axonemal intermediate chain 2	0.5	0.34
*DNAAF1*	dynein axonemal assembly factor 1	0.654	0.33
*ARMC4*	armadillo repeat containing 4	0.615	0.32
*DRC1*	dynein regulatory complex subunit 1	0.654	0.32
*FOXJ1*	forkhead box J1	0.577	0.32
*HYDIN*	HYDIN axonemal central pair apparatus protein	0.538	0.31
*TTC25*	tetratricopeptide repeat domain 25	0.538	0.31
*TEKT1*	tektin 1	0.308	0.01

Data retrieved from the Disgenet database (https://www.disgenet.org/)

*PCD* Primary Ciliary Dyskinesia

*DPI* Disease Pleiotropy index for the gene

Score: Gene-Disease Association Score

### Gene analysis by Venn diagram

**Fig. 7:**
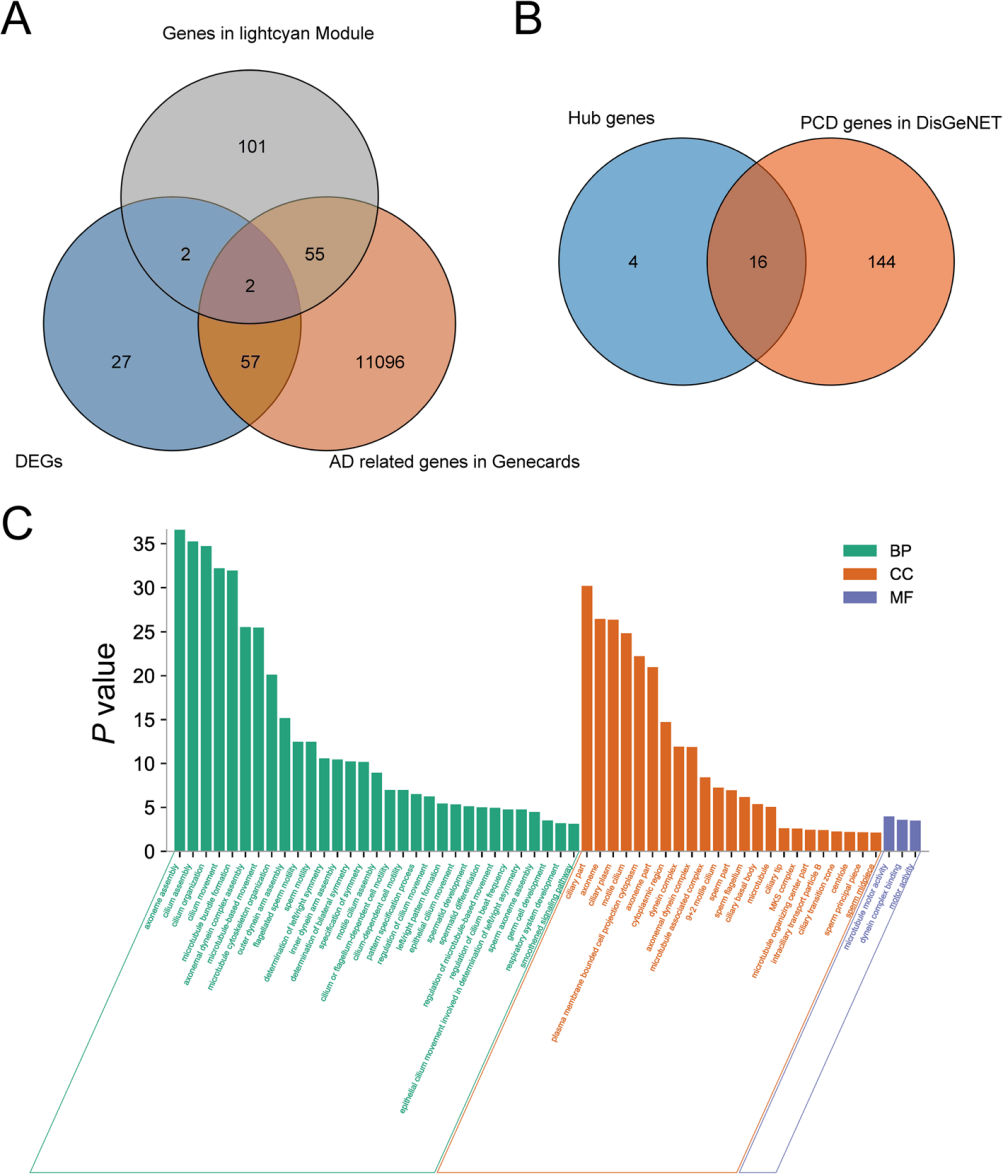
Venn diagram of different gene sets and Path analysis diagram of hub genes. **A** Venn diagram of DEGs showing genes in lightcyan Model and AD related genes in Genecards database. **B** Venn diagram of hub genes and PCD genes in Disgenet database. (C) GO analysis diagram of hub genes

In order to verify the reliability of the predicted results, we used the Venn diagram to intersect the DEGs, the related genes from WGCNA analysis, and the genes related to AD in Genecards database. The results showed that there were 59 common genes between DEGs and AD related genes in Genecards and 57 common genes between genes in lightcyan module and AD related genes in Genecards, indicating that the diagnostic efficiency of the two methods was comparable (Fig. 7A, B). Therefore, we used the method of WGCNA to identify the gene module related to AD and PPI to identify the hub genes. Then, we used Venn diagram to intersect the hub genes and the genes related to PCD in Disgenet database. The results showed that 16 out of the 20 hub genes were duplicated, indicating that these hub genes were closely related to PCD. In order to investigate the functions of these hub genes, we further annotated these genes based on GO and KEGG databases. Results of GO annotation showed that these genes were closely associated with the axoneme and cilium (Fig. 7C, Table S5). The results of the enrichment analysis based on KEGG database showed that these hub genes were closely related to Huntington’s disease, which shares many medical similarities with AD.

### Gene interaction heatmap

**Fig. 8:**
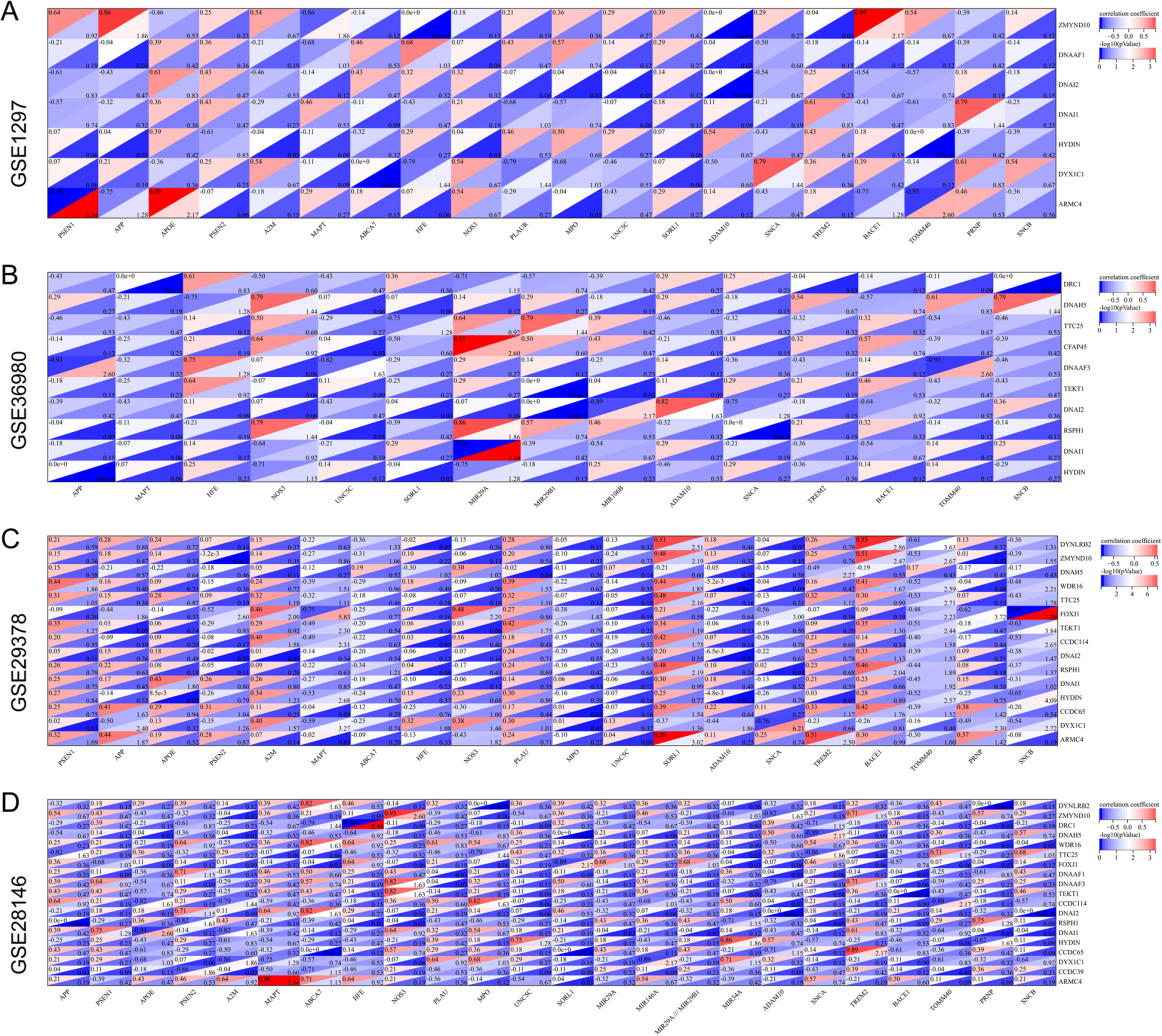
Gene interaction heatmap between hub genes and key genes related to AD. The hub genes identified (genes with incomplete information were deleted) were used as the vertical axis and the top 30 genes with the highest correlation with AD in Disgenet database was set as the horizontal axis. and the color bar represents correlation coefficients with red representing positive correlation, blue negative correlation, and darker color strong correlation. Asterisks represent levels of significance (* *p* < 0.05; ** *p* < 0.01). **A** Gene Interaction Heatmap of GSE1297. **B** Gene Interaction Heatmap of GSE36980. **C** Gene Interaction Heatmap of GSE29378. (D) Gene Interaction Heatmap of GSE28146

Because of the application of different chip platforms in investigating the expression of genes, many genes were not detected in GPL570 [HG-U133_Plus_2] Affymetrix Human Genome U133 Plus 2.0 Array. Therefore, the top 30 genes were selected and defined as the genes most closely related to AD. These results suggested that hub genes showed varied degrees of correlation with genes related to AD (Fig. 8; Table S6).

### Prediction of miRNAs of hub genes

**Fig. 9:**
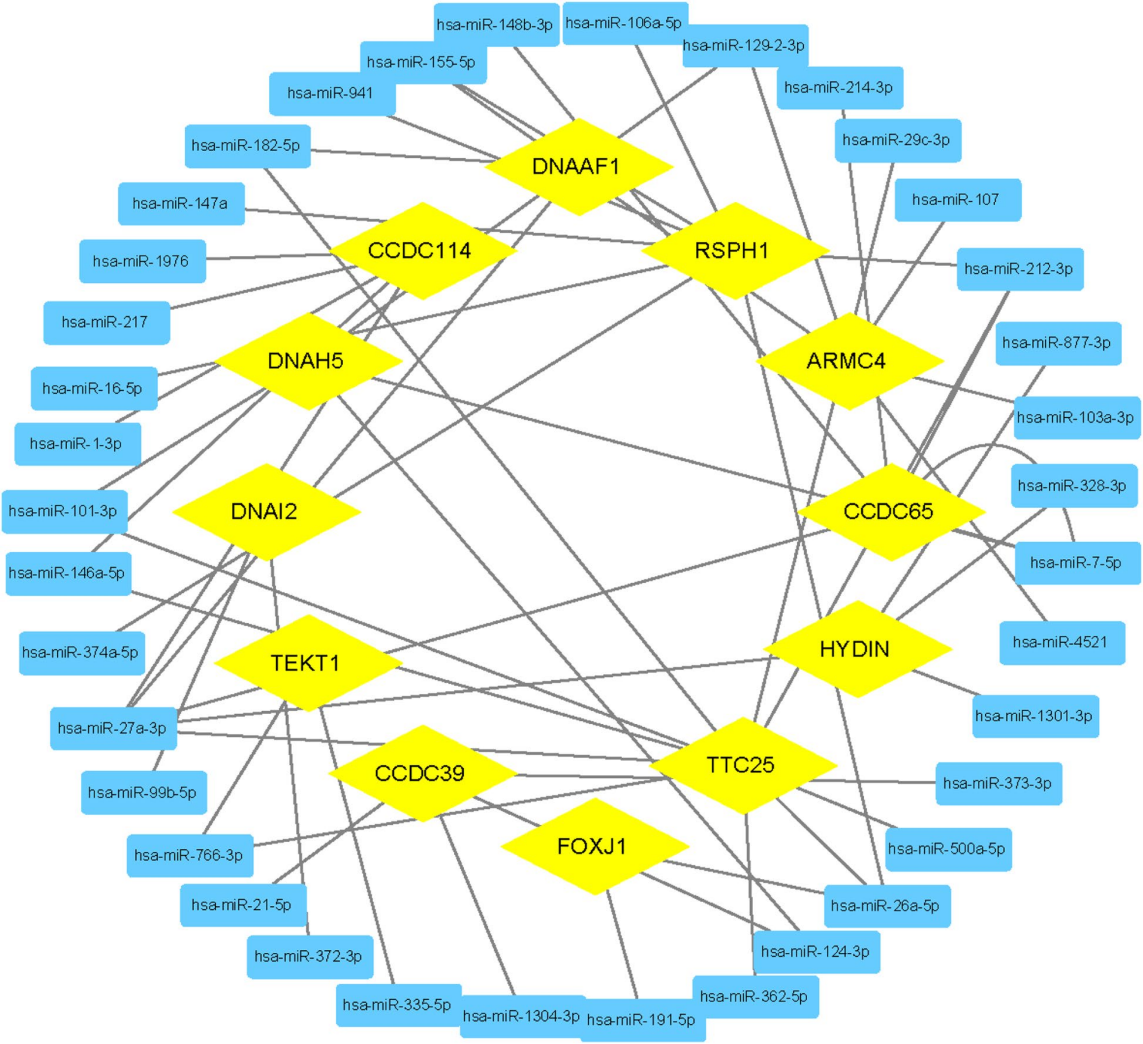
Prediction of miRNAs of hub genes related to AD. The yellow diamond in the figure represents the hub gene, and the blue rectangle represents the miRNA associated with it

After importing hubgenes into miRecords, we removed four genes that were not included in the website, and then we got 37 related miRNAs (Fig. 9).

## Discussion

As people’s average life expectancy has increased due to the further improvement of modern medicine and science, the number of patients with AD will be increased as well [38]. Genome-wide association studies have identified numerous genomic loci associated with AD, while the causal genes and variants are still being continuously identified [39]. Most of the conventional methods of differential gene analysis focus on the differential expression of a single gene, i.e., the greater the difference of a single gene expression, the more important the role the single gene plays [40]. In our study, we identify the genes with the highest changes in the expression in the hippocampus of AD patients. We further investigate these genes with functional enrichment analysis. Studies have shown that *LTF* can increase the α-Secretase-Dependent amyloid precursor protein processing via the ERK1/2-CREB and HIF-1α pathways in a mouse model of AD [41]. Furthermore, loss of *SLC17A6* is correlated with cognitive decline in AD [42]. Although cilia and flagella associated protein (CFAP) is essentially important for sperm flagellum biogenesis [43], no association has been revealed between *CFAP126* and AD. In the APP/PS1 mouse model of AD, showing anxiety-like behavior, the photo stimulating the pBLA-vCA1 circuit ameliorated the anxiety in a Calb1-dependent manner [44]. Similar to these genes, many of the DEGs identified in our study are related to AD with only one gene related to cilia. The KEGG functional enrichment analysis reveal that these DEGs are involved in pathways of nicotine addiction, neuroactive ligand-receptor interaction, vesicle-mediated transport in synapse, synaptic vesicle cycle, neurotransmitter transport, and synapse organization. The KEGG pathway of nicotine addiction is associated with neurological diseases [45], and other pathways are related to synapse, whereas no association with cilia has been identified.

GSEA uses the pre-defined gene set to rank the genes based on the degree of differential expression. This analysis has been used to identify key transcriptome biomarkers in AD [46]. The results of our GSEA analysis show that the genes have implications for disease are enriched in four gene sets, i.e., *CHR2Q13*, *CHR19P12*, *CHR4P14*, and *KRAS.PROSTATE_UP. V1_DN*. The differences of the three gene sets of *CHR2Q13*, *CHR19P12* and *CHR4P14* suggest that there may be lesions on chromosomes 2, 4 and 19 in the hippocampus of AD patients. The differences in the *KRAS.PROSTATE_UP.V1_DN.* gene set suggest that the changes of genes in the hippocampus of AD patients are similar to those of epithelial prostate cancer cell lines. The gene *TUBAL3* related to cilia is identified only in *KRAS.PROSTATE_UP. V1_DN*. AD is often referred to as an immortal cancer, and some studies suggest it is indeed linked to cancer, of 8097 AD patients, the HR (Hazard Ratio) for all subsequent cancers was 0.822 (95% CI, 0.728–0.928; *P* = 0.002). Among them, three specific cancers were associated with AD: lung cancer (HR, 0.656; 95% CI, 0.494–0.871; *P* = 0.004), prostate and testicular cancer (HR, 0.414; 95% CI, 0.202–0.847; *P* = 0.016), and lymphoma (HR, 2.202; 95% CI, 1.005–4.826; *P* = 0.049) [47]. We hope to find a link between the two diseases in this paper with this approach, but unfortunately, our idea failed. Then we analyze the reasons for this failure, mainly because PCD is a relatively rare disease, and it is not included in the disease-related gene set analyzed by GSEA. In the future, with the continuous improvement of this method, maybe our idea is likely to succeed.

With the development of bioinformatics technology, more and more advanced and adequate methods have been developed and applied in life sciences [48]. At present, WGCNA is a well-established method and applied in various studies of human diseases [28]. In our study, the results of the WGCNA analysis show that 12 modules are related to AD, and the lightcyan module showing the highest correlation with AD contained a total of 160 genes. Based on the PPI network, 20 hub genes are identified to play important roles in the network associated with PCD [49–52]. We rank the hub genes following the Score obtained in the DisGeNET database, and the results reveal that the top four genes include *DNAI1*, *DNAAF3*, *CCDC114*, and *CCDC65*. Studies have shown that *DNAI1* is strongly linked to the ciliary beat pattern variations [49], while the *DNAAF3* variation in respiratory cilia has been found uniformly immotile due to their defected dynein arms [50]. *CCDC114* is located at the basal body of a cilium and the knockdown of *CCDC114* could affect the formation of cilia in hRPE1 cells [51]. *CCDC65* is a central hub gene for assembly of the nexin-dynein regulatory complex and other regulators of ciliary and flagellar motility [52]. The results of both the GO and KEGG analyses show that these hub genes are not only related to the regulation of axonemal dynein complex assembly and cilium movement, but also play an important role in Huntington’s disease. It is worth noting that both the axonemal dynamic complex and cilium movement are relevant to neurological diseases [53].

Gene interaction heatmap shows that the hub genes are associated with AD related key genes. In such network, highly connected genes are called hub genes, which are expected to play an important role in understanding the biological mechanism of response under stresses/conditions. Gene interaction is used to predict the function of the unknown gene [54]. Our results indicate a relationship between PCD and AD. Although we found some connections between genes using sequencing results, the underlying reasons for these connections still need to be verified by experiments such as RT-PCR.

Many miRNAs have been used as tools for the diagnosis and treatment of AD [55], but miRNAs for PCD are rarely mentioned. We obtain these miRNAs through the prediction of hub gene. Since these genes are related to both AD and PCD, maybe these miRNAs It can be used for the diagnosis and treatment of these two diseases in the future.

The role of Aβ in AD development has been widely recognized with its deposition as one of the main symptoms of AD. Studies show that the Aβ inhibition of mitochondrial axonemal transport is associated with the early pathophysiology of AD [56–58]. Furthermore, it has been reported that the Aβ is transmitted through neuronal connections on the axon membrane [59]. PCD is a multiple inherited disorder caused by ciliary structural defects [60]. Evidently, PCD is directly related to ciliary movement. Moreover, studies have shown that the motor protein of axons is related to PCD [61, 62]. These results suggest that AD and PCD are linked by the functions of cilia and axons.

We have applied not only the conventional methods of differential gene analysis, but also the GSEA and WGCNA to analyze the experiment data and to identify the gene sets related to PCD in the hippocampus of AD patients. We have withdrawn the following conclusions. First, most genes obtained by conventional differential gene analysis have been confirmed by our results. The GO and KEGG analyses further verify that these genes are related to AD. However, both the enrichment and GSEA analyses fail to identify any association between AD and PCD. Second, PCD related modules in hippocampus of AD patients are discovered by WGCNA. Third, the gene interaction heatmap shows that hub genes are bound to AD related key genes. These results indicate that the hub genes are involved in the regulation of axonemal dynein complex assembly and the regulation of cilium movement, both of which played important roles in AD as well. These results strongly suggest that both AD and PCD were related in the functions of cilia and axons.

## Conclusion

Based on the results of establishing the key gene network through WGCNA, our findings suggest that AD and PCD may share the pathogenesis, mainly reflect in the functions of cilia and axons. These commonalities indicate strongly the association between AD and PCD, providing theoretical foundations for further exploration of the pathogenesis and treatment of these two human diseases.

## Supplementary Information

**Additional file 1: Figure S1**. The heatmap of gene sets *CHR2Q13* analyzed by GSEA.

**Additional file 2: Figure S2**. The heatmap of gene sets *CHR4P14* analyzed by GSEA.

**Additional file 3: Figure S3**. The heatmap of gene sets *CHR19P12* analyzed by GSEA.

**Additional file 4: Figure S4**. The heatmap of gene sets *KRAS.PROSTATE_UP.V1_DN* analyzed by GSEA.

**Additional file 5: Table S1**. Sequencing of hippocampal data in dataset GSE48350.

**Additional file 6: Table S2**. Differentially expressed genes of Table S1. (CSV 15813 kb)

**Additional file 7: Table S3**. GO and KEGG analysis of differentially expressed genes in Table S2.

**Additional file 8: Table S4**. Module info of WGCNA analysis.

**Additional file 9: Table S5**. GO analysis diagram of hub genes.

**Additional file 10: Table S6**. Gene Interaction Heatmap data.

## Data Availability

The raw data of this study are derived from the GEO data portal (https:// www. Ncbi. nlm. Nih. gov/ geo/), GSE48350/ GSE28146/GSE1297/GSE36980/GSE19378 which are publicly available databases.

## Abbreviations

AD: Alzheimer’s Disease
PCD: Primary Ciliary Dyskinesia
AHN: Adult Hippocampal Neurogenesis
DEGs: Differentially Expressed Genes
GEO: Gene Expression Omnibus
GO: Gene Ontology
KEGG: Kyoto Encyclopedia of Genes and Genomes
MEs: Module Eigengenes
MM: Module Membership
PPI: Protein-protein Interaction
WGCNA: Weighted Gene Co-expression Network Analysis
CTs: Control Samples
NES: Normalized Enrichment Score
FDR: False Discovery Rate
A-β: Amyloid β-protein
