# Supplementary figures and images for: Key gene network related to primary ciliary dyskinesia in hippocampus of patients with Alzheimer’s disease revealed by weighted gene co-expression network analysis
Source: BMC Neurol. 2022 May 30;22:198. doi: 10.1186/s12883-022-02724-z (PMC9150314; doi:10.1186/s12883-022-02724-z)

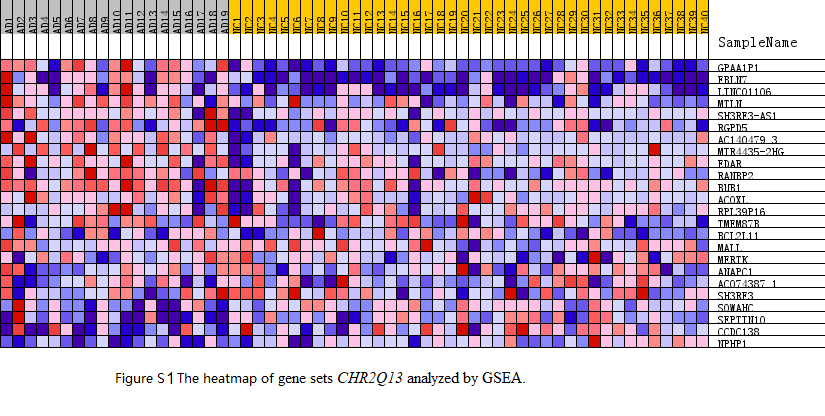

Supplement: Supplementary file 1 — Additional file 1: Figure S1. The heatmap of gene sets CHR2Q13 analyzed by GSEA. [file 12883_2022_2724_MOESM1_ESM.png]

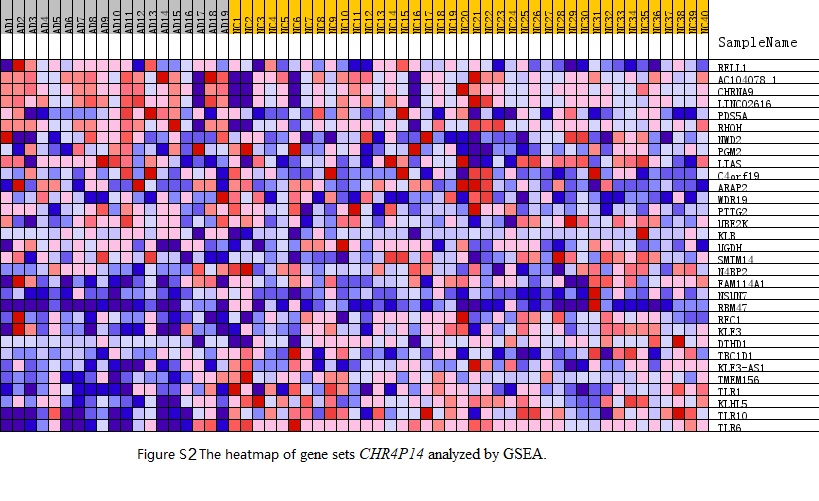

Supplement: Supplementary file 2 — Additional file 2: Figure S2. The heatmap of gene sets CHR4P14 analyzed by GSEA. [file 12883_2022_2724_MOESM2_ESM.png]

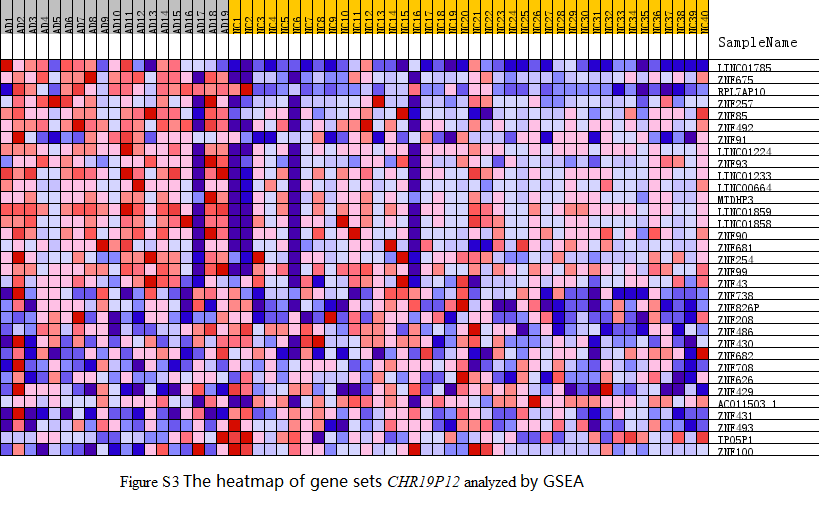

Supplement: Supplementary file 3 — Additional file 3: Figure S3. The heatmap of gene sets CHR19P12 analyzed by GSEA. [file 12883_2022_2724_MOESM3_ESM.png]

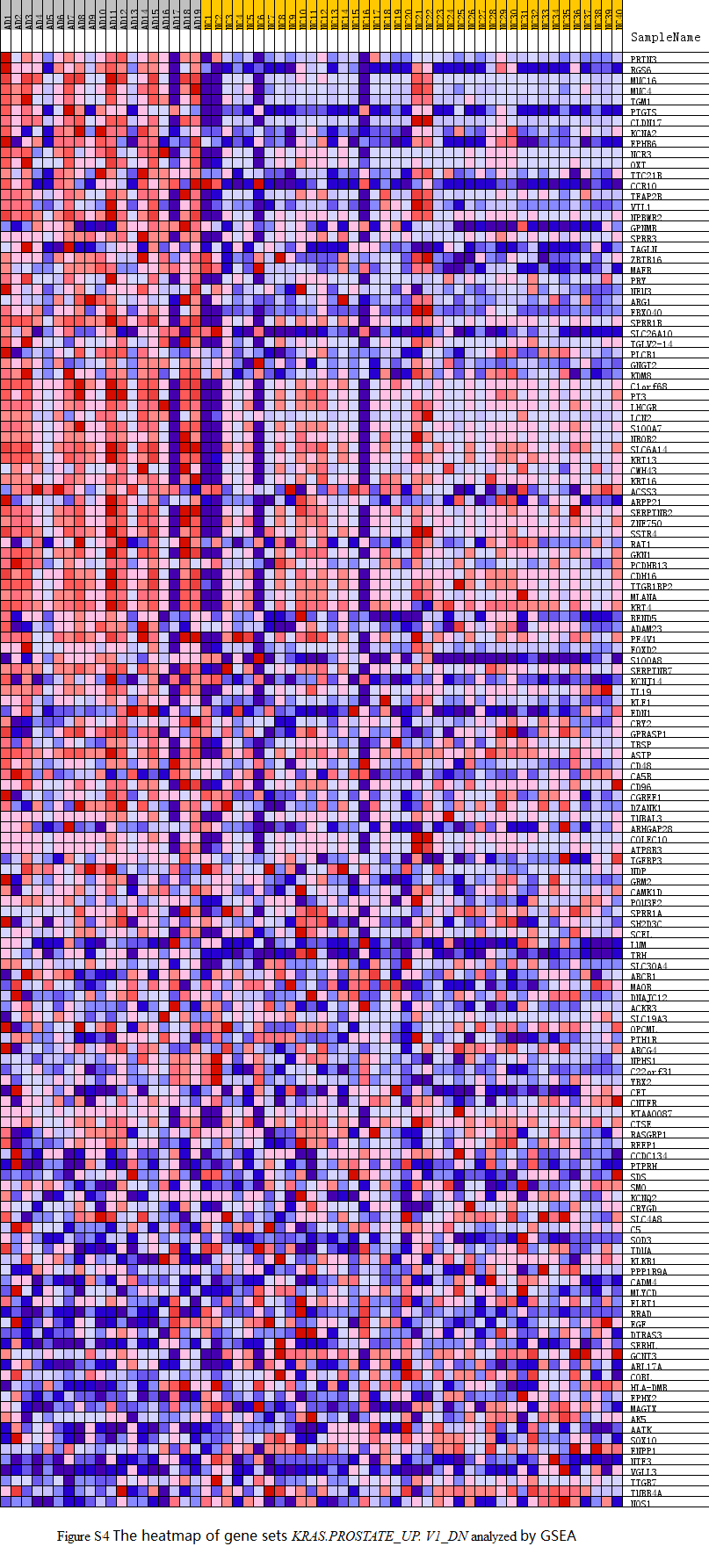

Supplement: Supplementary file 4 — Additional file 4: Figure S4. The heatmap of gene sets KRAS.PROSTATE_UP.V1_DN analyzed by GSEA. [file 12883_2022_2724_MOESM4_ESM.png]
